# Supplementary material for: GFVO: the Genomic Feature and Variation Ontology
Source: PeerJ. 2015 May 5;3:e933. doi: 10.7717/peerj.933 (PMC4435477; doi:10.7717/peerj.933)
Supplement: Supplemental Information 1 [file peerj-03-933-s001.docx]

## Supplementary Information

The following examples of GFVO applications are formatted in RDF Turtle. The examples are also available through the ontology’s GitHub repository — branch “master”, directory “gfvo_paper_2015” — as well as the BioInterchange ontology web-page.

Examples 1 and 2 address genomic features and relationships between them as they are described by the GFF3 specification. Example 1 also illustrates the encoding of genomic features as defined by the GTF specification, since the GFF3 specification subsumes the GTF specification. Examples 3, 3a and 4 focus on the representation of genomic feature attributes as they are described in the GVF specification. Example 5 shows a representation of VCF genomic features.

### Example 1: describing a gene and its transcription factor binding site (GFF3/GTF)

The GFF3 specification provides an exemplary description of a gene and its related genomic entities. Lines 0-3 were taken from the example in the GFF3 specification and translated into RDF Turtle using GFVO here; the used example excerpt from the GFF3 specification is:

##gff-version 3

##sequence-region ctg123 1 1497228

ctg123 . gene 1000 9000 . + . ID=gene00001;Name=EDEN

ctg123 . TF_binding_site 1000 1012 . + . ID=tfbs00001;Parent=gene00001

In the following, the GFF3 file contents are encapsulated via the named entity “ExampleSet1”. “ExampleSet1” is associated with information that spans the entirety of the GFF3 file, e.g. versioning information regarding the GFF3 file format. Genomic features that are encoded on separate lines in the GFF3 file become named entities “ExampleSet1Feature1” and “ExampleSet1Feature2”. Both features are located on the same landmark, “ctg123”, which is described via the named entity “ExampleSet1Landmark” and linked to both genomic features via their annotation with the object property “isLocatedOn”.

@prefix : <http://www.biointerchange.org/gfvo#> .

@prefix faldo: <http://biohackathon.org/resource/faldo#> .

@prefix so: <http://purl.bioontology.org/ontology/SO/SO> .

:ExampleSet1 a :File ;

:hasIdentifier

[

a :Version ;

:hasValue "gff-version 3" .

] ;

:hasMember :ExampleSet1Feature1 ,

:ExampleSet1Feature2 .

# Feature 1: a gene

:ExampleSet1Feature1 a :Feature ,

so:0000704 ;

:isLocatedOn :ExampleSet1Landmark ;

:hasPart

[

a :Locus ;

:hasAttribute

[

a faldo:Region ;

faldo:begin

[

a faldo:ExactPosition ;

faldo:position 1000 .

] ;

faldo:end

[

a faldo:ExactPosition ;

faldo:position 9000 .

] .

] .

] ;

:hasIdentifier

[

a :Identifier ;

:hasValue "gene00001" .

] ;

:hasAttribute

[

a :Name ;

:hasValue "EDEN" ;

:hasAttribute

[

a :Label ;

:hasValue "Name" .

] .

] .

# Feature 2: a transcription factor binding site

:ExampleSet1Feature2 a :Feature ,

so:0000235 ;

:isLocatedOn :ExampleSet1Landmark ;

:hasPart

[

a :Locus ;

:hasAttribute

[

a faldo:Region ;

faldo:begin

[

a faldo:ExactPosition ;

faldo:position 1000 .

] ;

faldo:end

[

a faldo:ExactPosition ;

faldo:position 1012 .

] .

] .

] ;

:hasIdentifier

[

a :Identifier ;

:hasValue "tfbs00001" .

] ;

:hasSource :ExampleSet1Feature1 .

# Features in this example are placed on a single landmark, which has

# an identifier associated with it (it cannot be anonymous in GFF3, even

# though GFVO permits landmarks without explicit identifier), and it

# has a range of sequence positions it covers (expressed by a separate

# pragma statement in GFF3).

:ExampleSet1Landmark a :Landmark ;

:hasIdentifier

[

a :Identifier ;

:hasValue "ctg123" .

] ;

:hasAttribute

[

a faldo:Region ;

faldo:begin

[

a faldo:ExactPosition ;

faldo:position 1 .

] ;

faldo:end

[

a faldo:ExactPosition ;

faldo:position 1497228 .

] .

] .

### Example 2: a sequence alignment (GFF3)

GFF3 files capture sequence alignments between two nucleotide or amino acid sequences in CIGAR format (cf. “The Gap Attribute”, GFF3 specification). The CIGAR format describes sequence alignments as an ordered list of alignment operations, each of which can range over a span of nucleotides/amino acids. This example is a translation of a CIGAR formatted sequence alignment of the GFF3 file format specification, namely:

chr3 . Match 1 23 . . . ID=Match1;Target=EST23 1 21;Gap=M8 D3 M6 I1 M6

The following RDF Turtle translation represents the CIGAR format based sequence alignment between the named entities “ExampleSet2Feature1” and “ExampleSet2Feature2” via the GFVO “SequenceAlignment” class instance “ExampleSet2SeqAlignment”. The latter references the operations that uniquely define the sequence alignment, which are subclasses of the GFVO class “SequenceAlignmentOperation”.

@prefix : <http://www.biointerchange.org/gfvo#> .

@prefix faldo: <http://biohackathon.org/resource/faldo#> .

@prefix so: <http://purl.bioontology.org/ontology/SO/SO> .

:ExampleSet2 a :Collection ;

:hasMember :ExampleSet2Feature1 ,

:ExampleSet2Feature2 .

:ExampleSet2Landmark a :Landmark ;

:hasIdentifier

[

a :Identifier ;

:hasValue "chr3" .

] .

# Reference sequence feature:

:ExampleSet2Feature1 a :Feature ;

:isLocatedOn :ExampleSet2Landmark ;

:hasIdentifier

[

a :Identifier ;

:hasValue "Match1" .

] .

# Target sequence feature:

:ExampleSet2Feature2 a :Feature ,

so:0000343 ;

:isLocatedOn :ExampleSet2Landmark ;

:hasIdentifier

[

a :Identifier ;

:hasValue "EST23" .

] .

# Description of the sequence alignment between :ExampleSet2Feature1

# and :ExampleSet2Feature2 (features "Match1" and "EST23").

:ExampleSet2SeqAlignment a :SequenceAlignment ;

:hasAttribute

[

a :Locus ;

:hasAttribute

[

a faldo:Region ;

faldo:begin

[

a faldo:ExactPosition ;

faldo:position 1 .

] ;

faldo:end

[

a faldo:ExactPosition ;

faldo:position 21 .

] .

] .

] ;

:hasSource :ExampleSet2Feature1 ;

:hasInput :ExampleSet2Feature2 ;

:hasFirstPart :ExampleSet2AlignmentOp1 ;

:hasOrderedPart :ExampleSet2AlignmentOp2 ,

:ExampleSet2AlignmentOp3 ,

:ExampleSet2AlignmentOp4 ;

:hasLastPart :ExampleSet2AlignmentOp5 .

# Details about the alignment operations: their type and sequence spans

:ExampleSet2AlignmentOp1 a :Match ;

:hasAttribute

[

a :Span ;

:hasValue 8 .

] ;

:isBefore :ExampleSet2AlignmentOp2 .

:ExampleSet2AlignmentOp2 a :TargetSequenceGap ;

:hasAttribute

[

a :Span ;

:hasValue 3 .

] ;

:isAfter :ExampleSet2AlignmentOp1 ;

:isBefore :ExampleSet2AlignmentOp3 .

:ExampleSet2AlignmentOp3 a :Match ;

:hasAttribute

[

a :Span ;

:hasValue 6 .

] ;

:isAfter :ExampleSet2AlignmentOp2 ;

:isBefore :ExampleSet2AlignmentOp4 .

:ExampleSet2AlignmentOp4 a :ReferenceSequenceGap ;

:hasAttribute

[

a :Span ;

:hasValue 1 .

] ;

:isAfter :ExampleSet2AlignmentOp3 ;

:isBefore :ExampleSet2AlignmentOp5 .

:ExampleSet2AlignmentOp5 a :Match ;

:hasAttribute

[

a :Span ;

:hasValue 6 .

] ;

:isAfter :ExampleSet2AlignmentOp4 .

### Example 3: encoding of phased genotypes and sequence variants (GVF)

GVF formatted files — besides VCF formatted files — are used to share data about genomic sequence variations. In this example, the GFVO representation for the GVF specification’s “Genotype” attribute illustration for multiple sequenced individuals are given:

Variant_seq=A,T;Genotype=0:1,1:1

These attributes were added to a minimal GVF file for the purpose of providing a valid reference file. Since this example addresses phased genotypes, the “##phased-genotypes” pragma statement was included in the GVF file. The reference defines a single variant sequence-alteration feature (Sequence Ontology accession SO:0001059), at an arbitrarily chosen genomic location (landmark “chr1”, genomic start-/end-coordinates at 1000, forward strand). Attributes “ID” and “Reference_seq” were included as required per GVF specification, where the value of “ID” was freely chosen and it was decided that “Reference_seq” should coincide with the first sequence variant (“A”, adenine). The contents of the minimal GVF file are:

##gvf-version 1.07

##phased-genotypes

chr1 n/a SO:0001059 1000 1000 . + . ID=Example3;Reference_seq=A;Variant_seq=A,T;Genotype=0:1,1:1

In the following, the named entity “ExampleSet3Genotype1” encodes a heterozygous genotype (A/T), whereas “ExampleSet3Genotype2” encodes a homozygous genotype (T/T). Phase of genotypes is denoted with the help of “SequencedIndividual” instances “ExampleSet3SequencedIndividual1” and “ExampleSet3SequencedIndividual2”; associating a genotype with the latter instances establishes the provenance of genomic material, and hence, phase. For brevity, information about genomic landmark, coordinates, identifier labeling, and reference sequence has been omitted.

@prefix : <http://www.biointerchange.org/gfvo#> .

:ExampleSet3 a :Collection ;

:hasMember :ExampleSet3Feature1 .

# The individuals that were sequenced are represented by named,

# but otherwise unspecified, instances. This provides sufficient

# information to distinguish between the individuals that were

# sequenced.

:ExampleSet3SequencedIndividual1 a :SequencedIndividual .

:ExampleSet3SequencedIndividual2 a :SequencedIndividual .

# Genomic feature that captures the two described genotypes.

:ExampleSet3Feature1 a :Feature ;

:hasAttribute :ExampleSet3Genotype1 ,

:ExampleSet3Genotype2 .

# Genotype for the first individual.

:ExampleSet3Genotype1 a :Genotype ;

:hasParticipant :ExampleSet3SequencedIndividual1 ;

:hasQuality :Heterozygous ,

:GameticPhase ;

:hasFirstPart :ExampleSet3ReferenceA_AT ;

:hasLastPart :ExampleSet3VariantT_AT .

# Ordering of the variants permits the expression of phased genotypes.

# It is recommended to use "isBefore", but the use of "isAfter" is

# optional.

:ExampleSet3ReferenceA_AT :isBefore :ExampleSet3VariantT_AT .

:ExampleSet3VariantT_AT :isAfter :ExampleSet3ReferenceA_AT .

# Genotype of the second individual.

:ExampleSet3Genotype2 a :Genotype ;

:hasParticipant :ExampleSet3SequencedIndividual2 ;

:hasQuality :Homozygous,

:GameticPhase ;

:hasFirstPart :ExampleSet3VariantT_TT ;

:hasLastPart :ExampleSet3VariantT_TT .

# As above: variant ordering to phase the genotype.

:ExampleSet3VariantT_TT :isBefore :ExampleSet3VariantT_TT ;

:isAfter :ExampleSet3VariantT_TT .

# For VCF data, the type can also be "ReferenceSequence", which

# denotes that the sequence is located on a reference genome. This

# would be indicated -- in a VCF file -- by a "0" in the GT field

# (e.g., "0/1" to indicate a genotype whose first allele is on the

# reference genome and the second allele is an alternative allele).

:ExampleSet3ReferenceA_AT a :ReferenceSequence ;

:hasValue "A" .

:ExampleSet3VariantT_AT a :SequenceVariant ;

:hasValue "T" .

:ExampleSet3VariantT_TT a :SequenceVariant ;

:hasValue "T" .

#### Example 3a: encoding of unphased genotypes (GVF)

If “”ExampleSet3Genotype1” from above were not phased, i.e. unphased and the pragma statement “##phased-genotypes” were omitted, it could be expressed as follows where the ordering of the genotypes variants is no longer necessary:

@prefix : <http://www.biointerchange.org/gfvo#> .

:ExampleSet3a a :Collection ;

:hasMember :ExampleSet3aFeature1 .

:ExampleSet3aSequencedIndividual1 a :SequencedIndividual .

# Genomic feature that captures an unphased genotype.

:ExampleSet3aFeature1 a :Feature ;

:hasAttribute :ExampleSet3aGenotype1 .

:ExampleSet3aGenotype1 a :Genotype ;

:hasParticipant :ExampleSet3aSequencedIndividual1 ;

:hasQuality :Heterozygous ;

:hasPart

[

a :ReferenceSequence ;

:hasValue "A" .

] ,

[

a :SequenceVariant ;

:hasValue "T" .

] .

### Example 4: expression of Phred quality scores (GVF)

The GVF file-format specification recommends the use of Phred scaled quality scores for annotating genomic features. Since the use of Phred scaled quality scores is not enforced, the “score” column (column 6) of GVF files should generally be annotated using the GFVO “Score” class. If it is known that the “score” column is expressed in terms of Phred quality scores, then the GFVO “PhredScore” class should be used instead. The following GVF serves as input to this example:

##gvf-version 1.07

chr1 n/a SO:0001059 1000 1000 38 + . ID=Example4;Reference_seq=A;Variant_seq=A,T

As beforehand, previously demonstrated GFVO representations are omitted; the RDF Turtle representation of the GVF file does not show genomic landmarks, loci, or other annotations of the genomic feature than the Phred quality score. A GFVO “PhredScore”-class instance denotes the actual Phred quality score associated with the genomic feature.

@prefix : <http://www.biointerchange.org/gfvo#> .

:ExampleSet4 a :Collection ;

:hasMember :ExampleSet4Feature1 .

# Interpreting "score" (column 6) of a GVF file as Phred score.

# Uses "PhredScore" instead of "Score" to denote the specific

# type of score being used.

:ExampleSet4Feature1 a :Feature ;

:hasAttribute

[

a :PhredScore ;

:hasValue 38 .

] .

### Example 5: encoding of sequence variants, samples, and data filters (VCF)

VCF file contents are similar to GVF file contents, but permit further annotation of genomic features using data filters (pass/fail criteria) as well as indication of membership in reference database such as dbSNP and HapMap. The following example VCF file contents were lifted from the VCF specification, where only the first two genomic features are included here:

##fileformat=VCFv4.2

##contig=<ID=20,length=62435964,assembly=B36,md5=f126cdf8a6e0c7f379d618ff66beb2da,species="Homo sapiens",taxonomy=x>

##INFO=<ID=NS,Number=1,Type=Integer,Description="Number of Samples With Data">

##INFO=<ID=DP,Number=1,Type=Integer,Description="Total Depth">

##INFO=<ID=AF,Number=A,Type=Float,Description="Allele Frequency">

##INFO=<ID=H2,Number=0,Type=Flag,Description="HapMap2 membership">

##FILTER=<ID=q10,Description="Quality below 10">

##FILTER=<ID=s50,Description="Less than 50% of samples have data">

##FORMAT=<ID=GT,Number=1,Type=String,Description="Genotype">

##FORMAT=<ID=GQ,Number=1,Type=Integer,Description="Genotype Quality">

##FORMAT=<ID=DP,Number=1,Type=Integer,Description="Read Depth">

##FORMAT=<ID=HQ,Number=2,Type=Integer,Description="Haplotype Quality">

#CHROM POS ID REF ALT QUAL FILTER INFO FORMAT NA00001 NA00002 NA00003

20 14370 rs6054257 G A 29 PASS NS=3;DP=14;AF=0.5;DB;H2 GT:GQ:DP:HQ 0|0:48:1:51,51 1|0:48:8:51,51 1/1:43:5:.,.

20 17330 . T A 3 q10 NS=3;DP=11;AF=0.017 GT:GQ:DP:HQ 0|0:49:3:58,50 0|1:3:5:65,3 0/0:41:3

Meta-information lines (i.e., lines beginning with “##”) provide extra information about attributes in VCF files, such as data types as well as expected number of assigned data items. Some of this information can be represented exclusively as individual named entities as shown below (e.g, “:ExampleSet5Filter1”), whereas other meta-information lines are only partially represented via their own named entities and the remainder of the information is associated directly with a GFVO “Feature” instance (e.g., “:ExampleSet5Info1”). The latter enforces a uniform model of representing genomic features and variations across the various file formats, since the following VCF data is structurally coinciding with the GVF Examples 3 and 4.

@prefix : <http://www.biointerchange.org/gfvo#> .

@prefix faldo: <http://biohackathon.org/resource/faldo#> .

@prefix so: <http://purl.bioontology.org/ontology/SO/SO> .

:ExampleSet5 a :File ;

:hasIdentifier

[

a :Version ;

:hasValue "VCFv4.2" .

] ;

:hasMember :ExampleSet5Feature1 ,

:ExampleSet5Feature2 .

#

# INFO section.

#

# Note: "Number" and "Type" parameters are implicitly used in the

# conversion to RDF. For example, "Float" types are encoded

# as RDF floating point entities, whereas "Integer" types

# become RDF integer entities.

# "ID" assignments are encoded on a per-feature basis, which

# is in line with the representation chosen for GFF3/GTF/GVF

# file contents. The type of "ID" assignments is a GFVO Label,

# since the actual content type is not an identifier in the

# sense of GFVO/SIO.

#

:ExampleSet5Info1 a :Comment ;

:hasValue "Number of Samples With Data" .

:ExampleSet5Info2 a :Comment ;

:hasValue "Total Depth" .

:ExampleSet5Info3 a :Comment ;

:hasValue "Allele Frequency" .

:ExampleSet5Info4 a :Comment ;

:hasValue "HapMap2 membership" .

#

# FILTER section.

#

# Note: Filters are GFVO processes, i.e. "Process" sub-class instances.

# As before, "ID" assignments are represented by GFVO "Label"

# instances.

#

:ExampleSet5Filter1 a :VariantCalling ;

:hasAttribute

[

a :Label ;

:hasValue "q10" .

] ,

[

a :Comment ;

:hasValue "Quality below 10" .

] ,

[

a :Score ;

xsd:restriction

[

xsd:maxExclusive 10 .

] .

] .

:ExampleSet5Filter2 a :VariantCalling ;

:hasAttribute

[

a :Label ;

:hasValue "s50" .

] ,

[

a :Comment ;

:hasValue "Less than 50% of samples have data" .

] .

#

# FORMAT section.

#

# Note: "Number" and "Type" parameters are implicitly used in the

# conversion to RDF. For example, "String" types are encoded

# as RDF string entities, whereas "Integer" types become

# RDF integer entities.

#

# The expression "less than 50%" cannot be expressed using XML Schema.

# If a formal description is required, then it is suggested to use

# an RDF representation of MathML for expressing "less than 50%".

:ExampleSet5Format1 a :Comment ;

:hasValue "Genotype" ;

:hasAttribute

[

a :Identifier ;

:hasValue "GT" .

] .

:ExampleSet5Format2 a :Comment ;

:hasValue "Genotype Quality" ;

:hasAttribute

[

a :Label ;

:hasValue "GQ" .

] .

:ExampleSet5Format3 a :Comment ;

:hasValue "Read Depth" ;

:hasAttribute

[

a :Label ;

:hasValue "DP" .

] .

:ExampleSet5Format4 a :Comment ;

:hasValue "Haplotype Quality" ;

:hasAttribute

[

a :Label ;

:hasValue "HQ" .

] .

#

# Sample labels.

#

# Sample labels are taken from columns 10 and above of the "#CHROM" line.

#

:ExampleSet5Sample1 a :BiologicalEntity ;

:hasAttribute

[

a :Identifier ;

:hasValue "NA00001" .

] .

:ExampleSet5Sample2 a :BiologicalEntity ;

:hasAttribute

[

a :Identifier ;

:hasValue "NA00002" .

] .

:ExampleSet5Sample3 a :BiologicalEntity ;

:hasAttribute

[

a :Identifier ;

:hasValue "NA00003" .

] .

#

# "contig" line; definition of a genomic landmark.

#

:ExampleSet5Contig20 a :Landmark ,

so:0000340 ;

:isPartOf

[

a :Genome ;

:hasValue "B36" .

] ;

:hasAttribute

[

a :Identifier ;

:hasValue "20" .

] ,

[

a faldo:Region ;

faldo:begin

[

a faldo:ExactPosition ;

faldo:position 1 .

] ;

faldo:end

[

a faldo:ExactPosition ;

faldo:position 62435964 .

] .

] ,

[

a :InformationContentEntity ;

:hasValue "f126cdf8a6e0c7f379d618ff66beb2da" ;

:hasAttribute

[

a :Label ;

:hasValue "md5" .

] .

] ,

[

a :Reference ;

:hasValue "Homo sapiens" ;

:refersTo <http://www.ncbi.nlm.nih.gov/Taxonomy/Browser/wwwtax.cgi?id=9606> ;

:hasAttribute

[

a :Label ;

:hasValue "species" .

] .

] .

#

# Genomic feature descriptions.

#

:ExampleSet5Feature1 a :Feature ;

:isLocatedOn :ExampleSet5Contig20 ;

:hasPart

[

a :Locus ;

:hasAttribute

[

a faldo:ExactPosition ;

faldo:position 14370 .

] .

] ;

:hasIdentifier

[

a :Identifier ;

:hasValue "rs6054257" .

] ;

:hasAttribute

[

a :ReferenceSequence ;

:hasValue "G" .

] ,

[

a :SequenceVariant ;

:hasValue "A" ;

:hasAttribute

[

a :PhredScore ;

:hasValue 29 .

] .

] ,

[

a :SampleCount ;

:hasValue 3 ;

:hasAttribute

[

a :Name ;

:hasValue "NS" .

] .

] ,

[

a :Coverage ;

:hasValue 14 ;

:hasAttribute

[

a :Name ;

:hasValue "DP" .

] .

] ,

[

a :AlleleFrequency ;

:hasValue 0.5 ;

:hasAttribute

[

a :Name ;

:hasValue "AF" .

] .

] ,

[

a :ExternalReference ;

:refersTo <http://www.ncbi.nlm.nih.gov/SNP> ;

:hasAttribute

[

a :Name ;

:hasValue "DB" .

] .

] ,

[

a :ExternalReference ;

:refersTo <http://hapmap.ncbi.nlm.nih.gov> ;

:hasAttribute :ExampleSet5Info4 .

] ;

:hasEvidence

[

:hasSource :ExampleSet5Sample1 ;

:hasAttribute

[

a :Genotype ;

:hasQuality :Homozygous ,

:GameticPhase ;

:hasAttribute

[

a :ConditionalGenotypeQuality ;

:hasValue 48 .

] ,

[

a :Coverage ;

:hasValue 1 .

] ,

[

a :Haplotype ;

:hasFirstPart

[

a :PhredScore ;

:hasValue 51 .

] ;

:hasLastPart

[

a :PhredScore ;

:hasValue 51 .

] .

] ;

:hasFirstPart

[

a :ReferenceSequence ;

:hasValue "G" .

] ;

:hasLastPart

[

a :ReferenceSequence ;

:hasValue "G" .

] .

] ;

] ,

[

:hasSource :ExampleSet5Sample2 ;

:hasAttribute

[

a :Genotype ;

:hasQuality :Heterozygous ,

:GameticPhase ;

:hasAttribute

[

a :ConditionalGenotypeQuality ;

:hasValue 48 .

] ,

[

a :Coverage ;

:hasValue 8 .

] ,

[

a :Haplotype ;

:hasFirstPart

[

a :PhredScore ;

:hasValue 51 .

] ;

:hasLastPart

[

a :PhredScore ;

:hasValue 51 .

] .

] ;

:hasFirstPart

[

a :SequenceVariant ;

:hasValue "A" .

] ;

:hasLastPart

[

a :ReferenceSequence ;

:hasValue "G" .

] .

] ;

] ,

[

:hasSource :ExampleSet5Sample3 ;

:hasAttribute

[

a :Genotype ;

:hasQuality :Homozygous ;

:hasAttribute

[

a :ConditionalGenotypeQuality ;

:hasValue 43 .

] ,

[

a :Coverage ;

:hasValue 5 .

] ;

:hasPart

[

a :SequenceVariant ;

:hasValue "A" .

] ,

[

a :SequenceVariant ;

:hasValue "A" .

] .

] .

] .

:ExampleSet5Feature2 a :Feature ;

:isLocatedOn :ExampleSet5Contig20 ;

:hasPart

[

a :Locus ;

:hasAttribute

[

a faldo:ExactPosition ;

faldo:position 17330 .

] .

] ;

:isRefutedBy :ExampleSet5Filter1 ;

:hasAttribute

[

a :ReferenceSequence ;

:hasValue "T" .

] ,

[

a :SequenceVariant ;

:hasValue "A" ;

:hasAttribute

[

a :PhredScore ;

:hasValue 3 .

] .

] ,

[

a :SampleCount ;

:hasValue 3 ;

:hasAttribute

[

a :Name ;

:hasValue "NS" .

] .

] ,

[

a :Coverage ;

:hasValue 11 ;

:hasAttribute

[

a :Name ;

:hasValue "DP" .

] .

] ,

[

a :AlleleFrequency ;

:hasValue 0.017 ;

:hasAttribute

[

a :Name ;

:hasValue "AF" .

] .

] ;

:hasEvidence

[

:hasSource :ExampleSet5Sample1 ;

:hasAttribute

[

a :Genotype ;

:hasQuality :Homozygous ,

:GameticPhase ;

:hasAttribute

[

a :ConditionalGenotypeQuality ;

:hasValue 49 .

] ,

[

a :Coverage ;

:hasValue 3 .

] ,

[

a :Haplotype ;

:hasFirstPart

[

a :PhredScore ;

:hasValue 58 .

] ;

:hasLastPart

[

a :PhredScore ;

:hasValue 50 .

] .

] ;

:hasFirstPart

[

a :ReferenceSequence ;

:hasValue "T" .

] ;

:hasLastPart

[

a :ReferenceSequence ;

:hasValue "T" .

] .

] .

] ,

[

:hasSource :ExampleSet5Sample2 ;

:hasAttribute

[

a :Genotype ;

:hasQuality :Heterozygous ,

:GameticPhase ;

:hasAttribute

[

a :ConditionalGenotypeQuality ;

:hasValue 3 .

] ,

[

a :Coverage ;

:hasValue 5 .

] ,

[

a :Haplotype ;

:hasFirstPart

[

a :PhredScore ;

:hasValue 65 .

] ;

:hasLastPart

[

a :PhredScore ;

:hasValue 3 .

] .

] ;

:hasFirstPart

[

a :SequenceVariant ;

:hasValue "T" .

] ;

:hasLastPart

[

a :ReferenceSequence ;

:hasValue "A" .

] .

] .

] ,

[

:hasSource :ExampleSet5Sample3 ;

:hasAttribute

[

a :Genotype ;

:hasQuality :Homozygous ;

:hasAttribute

[

a :ConditionalGenotypeQuality ;

:hasValue 41 .

] ,

[

a :Coverage ;

:hasValue 3 .

] ;

:hasPart

[

a :SequenceVariant ;

:hasValue "T" .

] ;

:hasPart

[

a :SequenceVariant ;

:hasValue "T" .

] .

] .

] .
